# Early childhood development and urban environment in Mexico

**DOI:** 10.1371/journal.pone.0259946

**Published:** 2021-11-17

**Authors:** Francisco-Javier Prado-Galbarro, Carolina Pérez-Ferrer, Ana Ortigoza, Nancy Paulina López-Olmedo, Ariela Braverman-Bronstein, Rosalba Rojas-Martínez, Filipa de Castro, Tonatiuh Barrientos-Gutiérrez

**Affiliations:** 1 Population Health Research Center, National Institute of Public Health, Cuernavaca, Mexico; 2 Urban Health Collaborative, Drexel University, Philadelphia, PA, United States of America; The University of Hong Kong, HONG KONG

## Abstract

**Background:**

Childhood is considered the most important phase of human development; within it the period from birth to 5 years of age is particularly critical, given the speed at which changes occur. The context where children live can influence early childhood developmnent (ECD) by providing or limiting opportunities to learn, play and establish social interactions. This study explored the associations between characteristics of the urban environment and ECD in 2,194 children aged 36 to 59 months living in urban municipalities in Mexico

**Methods:**

We obtained ECD information from the 2015 Survey of Boys, Girls, and Women (ENIM, for its Spanish acronym), measured with the Early Childhood Development Index. The urban environment was evaluated at the municipal level, considering variables from five environment domains: physical, social, service, socioeconomic, and governance. Multilevel logistic models were fitted to assess the association between urban environment characteristics and the inadequacy of ECD in general and by specific development domains: learning, socio-emotional, physical, and alpha-numeric.

**Results:**

Inadequate ECD was inversely associated with the availability of libraries (OR = 0.55, 95% CI: 0.43, 0.72), and positively associated with population density (OR = 1.01, 95% CI: 1.01–1.02). For the specific ECD domains, inadequate socio-emotional development was inversely associated with the availability of libraries (OR = 0.66, 95% CI: 0.51, 0.85). Inadequate literacy-numeracy knowledge was associated inversely with the availability of daycare centers (OR = 0.56, 95% CI: 0.32, 0.97), and directly associated with the number of hospitals and clinics (OR = 1.87, 95% CI: 1.29, 2.72). Finally, the marginalization index was positively associated with inadequacy in the learning domain (OR = 1.80, 95% CI: 1.06, 3.03).

**Conclusions:**

Some aspects of the urban environment associated with ECD, suggest that intervening in the urban context could improve overall child development. Investment in resources oriented to improve socio-emotional development and literacy (such as libraries and daycare), could foster ECD in Mexico.

## Introduction

Child development involves all physical, socio-emotional, and cognitive processes that occur from pregnancy until youth, through which human beings progress towards increasing autonomy [1]. During the first five years, the brain architecture is shaped rapidly and major neurological achievements occur as a result of the interaction between the living context and the genetic inheritance [1–3]. This period of early child development (ECD) has a direct impact on lifetime trajectories and adult health [4–6]. Currently, more than 200 million children under 5 years from low- and middle-income countries (LMIC) are at risk of not achieving their full developmental potential [7].

Several studies have shown that ECD is influenced by individual, maternal, family, and school characteristics [6, 8]. However, distal determinants such as community or neighborhood characteristics have been scarcely studied [8, 9]. Recently, neighborhood urban environments were proposed as modifiable and potentially relevant factors for ECD [6, 10]. The presence of child relevant destinations, such as recreational centers, libraries, schools, or child health centers have been associated with better child development; also, parental perception of neighborhood safety was associated with better socioemotional development and overall health [11, 12]. Social conditions at the neighborhood level, such as social stressors and insecurity, have been hypothesized to influence ECD through maternal stress and fewer socialization opportunities for children [13].

**Fig 1:**
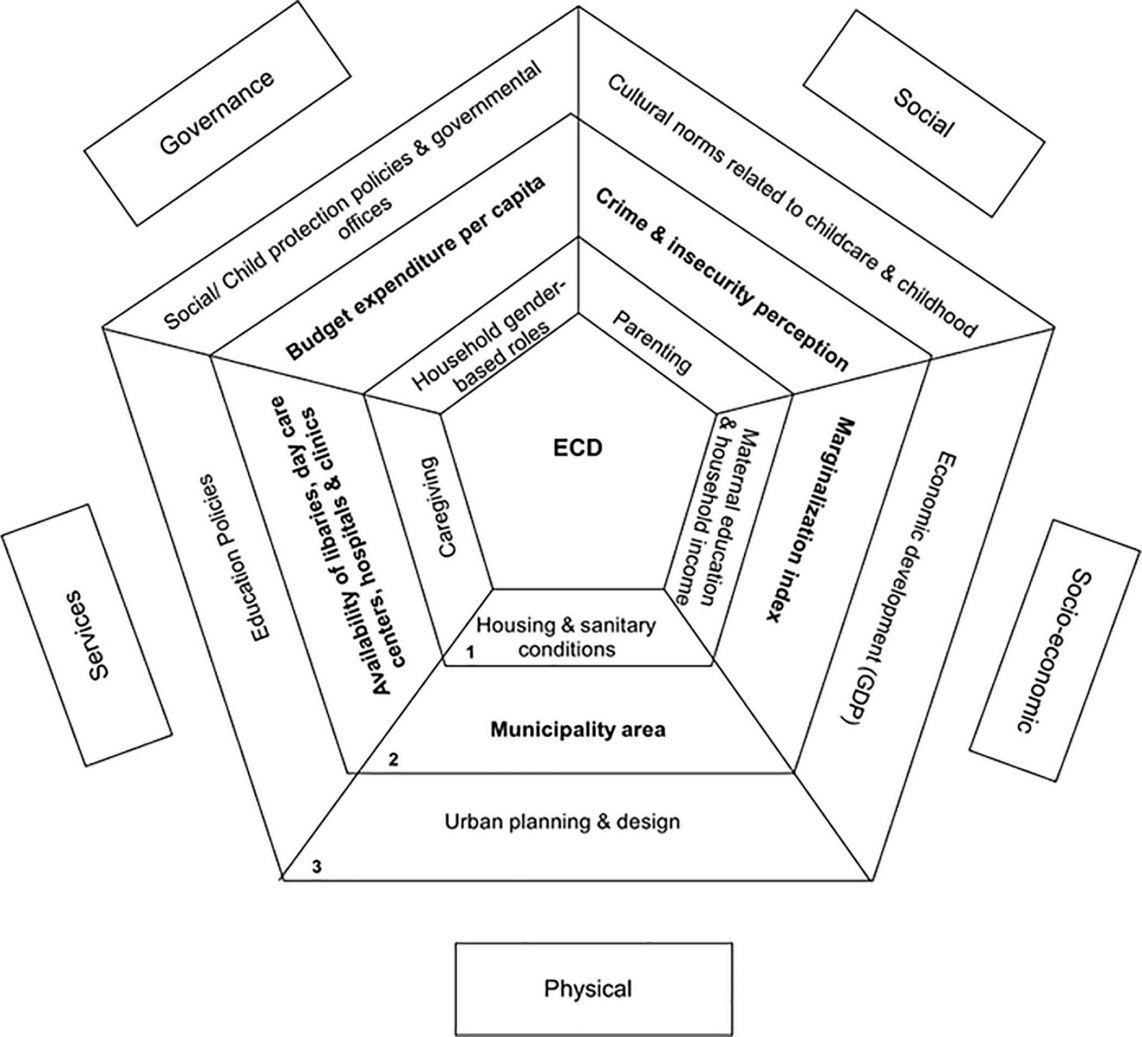
Conceptual model for the influence of neighborhood characteristics over early childhood development. (A) Outside boxes (and area below them) correspond to the environmental domains described by Goldfeld et al. Each domain encompasses variables at different levels represented by rows of the pentagon: 1 = household; 2 = municipality; 3 = country/structural context and corresponding measures influencing early child development (ECD). Bolded variables are the ones included in our study, aimed at assessing municipality features that could be associated with ECD. (B) Domains and levels are interconnected and influence overall ECD. For example, under governance domain, policies related to social protection could influence the way budget is spent at national and municipal level (proxied by budget per capita in our study). The way expenditures are assigned to women and children through social protection programs could influence women’s ability to decide household expenditures (household gender-based roles) and have agency on caregiving and parenting which could impact on child development through proper child stimulation by early schooling or at-home reading and playing.

Different frameworks have been proposed to explore the connection between the urban neighborhood environment and ECD [14, 15]. Goldfeld and colleagues [16] identified five domains related to ECD, 1) physical environment, such as parks, transportation, housing, and other conditions of the built environment [17]; 2) social environment, such as crime, trust, and safety; 3) socioeconomic conditions that include poverty, employment, and education, among others; 4) services, which include institutional resources such as schools, daycare centers, or healthcare facilities; and 5) governance, which refers to citizen engagement, leadership, and social coordination [16]. These upstream determinants of ECD are closely related to household and individual determinants, intervening as a whole by either supporting or constraining the environment for ECD, as presented in Fig 1.

The available evidence of the potential influence of urban environments on ECD is largely limited to high-income countries [6, 8–10]. Mexico is a middle-income, highly urbanized country, where rapid urbanization has led to poor urban planning, unequal distribution of services and resources, and large socioeconomic inequalities. This study aims to explore how the social and built environment in urban municipalities are associated with overall ECD and its specific domains in Mexico. We hypothesize that children living in municipalities with more crime and perception of insecurity (social domain) and higher marginalization, unemployment rate, and population density (socioeconomic domain) would experience higher odds of inadequate ECD. On the other hand, children in larger municipalities (physical domain) with more services (libraries, daycare centers, hospitals, and clinics), and better governance (more public budget spent and political corruption), would have better ECD outcomes.

## Methods

Data came from the National Survey of Children and Women (ENIM, for its Spanish acronym) which is a probabilistic survey with multi-stage, stratified, and cluster sampling, nationally representative of the rural and urban strata and five geographic regions, implemented as part of the 5^th^ round of the UNICEF’s (United Nations Children’s Fund) MICS (Multiple Indicator Cluster Surveys) Programme. The survey obtains information from four questionnaires: 1) household answered by the head of the household, 2) women aged 15 to 49 years, 3) children and adolescents aged 5 to 17 years old, answered by their mothers/primary caretakers, and 4) children under 5 years, answered by their mothers/primary caretakers [18]. For this study we used information from the household and the questionnaire for children under 5 years of age.

Only children aged 36 to 59 months old were eligible for the Early Childhood Development module of the survey, therefore, our study focuses on this age group. There were a total of 3,264 children aged 36 to 59 months old with complete questionnaires (98.2% of the sample in this age group) and we eliminated 1,070 living in rural localities, which left us with a final sample of 2,194 children who lived in 201 urban localities belonging to 192 municipalities. Urban localities are defined as localities with more than 2,500 inhabitants by the National Institute of Statistics and Geography [19]. Based on the child’s place of residence, we linked individual-level with municipal-level data using unique municipality codes. Municipalities are second-level administrative divisions (states being the first). They have legislative and executive authority and are responsible for the provision of basic public services for their population. On average, there were 17 children per municipality. All data used in this study is publicly available through Institutional websites.

### Outcome variable: Early childhood development

Early Childhood Development is a challenging construct to measure at the population level. For this purpose, UNICEF’s MICS Programme developed and validated the Early Childhood Development Index (ECDI) [20, 21]. From 2009 to 2019 the ECDI was adapted and implemented in more than 80 countries, making it the largest source of internationally comparable ECD outcomes in low- and middle-income countries. Despite limitations associated with data collection in large household surveys and its concise nature, studies indicate that the ECDI is useful for identifying inadequate development in children aged 36–59 months at the population level [22, 23].

The ECDI module explores four domains of development (literacy-numeracy, physical, socio-emotional, and learning), each one containing a number of items answered yes/no by the child’s primary caregiver to assess developmental adequacy; for this analysis we focused in children with inadequate development. For literacy-numeracy (3 items), development was considered to be adequate if a child could do at least two of the following: identify or name at least ten letters of the alphabet, read at least four common simple words, or recognize the symbols of all the numbers from 1 to 10. Adequate socio-emotional development (2 items) was obtained with at least two affirmative answers for the child gets along well with other children, does not kick, bite or hit adults or other children, and does not get distracted easily. Physical development (2 items) was considered adequate if a child could pick up small objects (rock, stick) with two fingers from the ground, and/or if the mother/primary caregiver indicated that the child was well enough to play. A child had adequate learning (2 items) if he/she could follow simple instructions on how to do things, and/or if he/she could independently follow an instruction given by an adult. To explore domain-specific associations, each domain was used as an independent binary outcome. We also classified adequacy of overall development following UNICEF guidelines, where a child is considered to have adequate ECD if he/she meets the age-expected development targets in at least three out of four domains [20].

### Exposure variables of the urban environment

Several urban conditions were considered key explanatory variables following the conceptual model by Goldfeld, et al. [16], which identifies five interconnected environment domains (as described in Fig 1): physical, social, service, socioeconomic, and governance. Exposure variables were all measured at the municipal level.

Social conditions were assessed using the prevalence of crime and the perception of insecurity. Both measures were obtained from the National Survey of Victimization and Perception of Public Safety (ENVIPE, for its Spanish acronym) [24]. The prevalence of crime indicates the proportion of people in each municipality who were victims of crime during 2016 (victims of multiple crimes were counted once). Perception of insecurity was defined as the proportion of adults who felt insecure in their municipality.

Physical conditions were assessed with proxies for urban growth, measures of the size and density of the area. Urban growth has been proposed as a relevant factor for health and wellbeing processes in cities [25, 26]. The municipal area corresponds to the total surface area of the municipality in km^2^, according to the National Geostatistical Framework [27]. Overall population density and population density of children under 5 years of age were calculated at the municipal level. This, using the total and specific population at each municipality in the year 2015 divided over the municipal area, as estimated by the National Institute of Statistic and Geography (INEGI, for its Spanish acronym) (inhabitants per km^2^) [28].

Services available in each municipality, specifically libraries, daycare centers, hospitals, and clinics were obtained from the National Statistical Directory of economic units (DENUE, for its Spanish acronym) derived from the Economic Censuses [29]. DENUE is an inventory of five million non-itinerant economic units related to manufacturing, commerce, and services. We obtained the number of preschool centers in the municipalities from the Census of Schools, Teachers, and Students of Basic and Special Education (CEMABE, for its Spanish acronym) [30]. These variables were standardized to z-scores; therefore, the analyses explore the odds of inadequate ECD associated with a one standard deviation change in the different services.

Socioeconomic conditions were captured using the marginalization index and the unemployment rate. The marginalization index was calculated by the National Population Council (CONAPO, for its Spanish acronym). It ranks municipalities based on education, housing quality, distribution of the population, and income, generating a continuous variable (range -2.22 to 1.39) [31]. The unemployment rate represented the proportion of people in a municipality who belonged to the economically active population and were not currently working, but were looking for work. Information was obtained from the National Occupation and Employment Survey [32].

Governance was captured using the per capita public budget spent during 2016 in Mexican pesos, an indicator obtained from the National Census of Transparency, Access to Public Information, and Protection of State Personal Data 2016 [33]. As a measure of political corruption, we used the number of administrative sanctions given by the state government to the municipality as a result of administrative negligence, failure to declare patrimonial assets, violation of laws, or nepotism. We obtained this information from the National Census of Municipal and Delegation Governments [34].

### Covariates

Individual characteristics of children such as sex, age in months, presence of functional difficulties, maternal age and education, indigenous condition, and wealth quintiles for urban households, were included as covariates. A child was considered to have functional difficulty if he/she experienced problems in at least one of the following areas: seeing, hearing, walking, fine motor, communication, learning, playing, or behavior. Maternal education was considered as the maximum level of school attendance achieved by the mother (none, primary, secondary, high school, or college and higher). Maternal age was categorized into five groups: 15–19, 20–24, 25–29, 30–34, 35–39, 40–44, and 45–49 years old. To adjust for household wealth we used the ENIM composite indicator, which includes household assets and characteristics. Households were classified into 5 categories, from the lowest (the poorest) to the highest quintile of the index (the richest) [18].

### Statistical analysis

We described the individual and municipal characteristics by ECD, using means for continuous variables and percentages for categorical variables. Bivariate analyses were conducted to determine the association between individual or municipal variables and inadequate ECD and each of its domains. A final model for each outcome was fitted including all significant urban environment variables from bivariate models. *One at a time*, *excluded variables were reintroduced in the model to assess their association in the multivariable environment*, *variables that became significant or that changed the coefficient of municipal variables by more than 10% were maintained in the model*. All models were adjusted by the same set of individual-level covariates: sex, age in months, functional disability, maternal education, wealth quintiles for urban households, indigenous condition, and maternal age. The association of urban environment and overall ECD and its domains was analyzed by fitting multilevel logistic regression with children clustered within municipalities:

$$y_{ij}∼\mathrm{Binomial}(1,\;\pi_{ij})$$

$$\log(\frac{\pi_{ij}}{1-\pi_{ij}})=\beta_{0}+\sum_{k=1}^{n}\beta_{k}X_{ki}+\sum_{l=1}^{m}\beta_{l}Z_{lj}+u_{j}$$

Where y_ij_ is the probability of inadequate ECD for child *i* in the municipality *j*. X_i_ is the set of explanatory variables at the individual level (level 1), and Z_j_ is the set of explanatory variables defined for the municipalities (level 2). u_j_ are the residuals of level 2, for which it is assumed that they are independent and follow a normal distribution with mean 0 and variance $\sigma_{u}^{2}$.

A null model was fitted to estimate the Median Odds Ratio (MOR), which is a measure of the variation of inadequate ECD across municipalities that is not explained by the modeled risk factors. Specifically, the MOR is the median value of odds ratios resulting from comparing pairs of children with identical individual-level covariates, chosen randomly from municipalities with different prevalence of inadequate ECD, and ordered so that the odds ratio is always at least one [35], obtained with the following formula:

$$MOR=\exp\left[\sqrt{\left(2*V_{A}\right)}*0.6745\right]\approx exp(0.95\sqrt{V_{A}})$$

V_A_ is the municipality-level variance, and the value 0.6745 is the 75th centile of the standard normal density. If the MOR is 1, there is no variation between municipalities; a larger MOR indicates considerable between-municipality variation.

All analyses considered the complex sampling design by applying the sampling weights provided by the ENIM and were conducted using Stata 14.0 (StataCorp, Stata Statistical Software, 2015).

## Results

The characteristics of the study population are summarized in Table 1. The mean age was 48.8 months (SD = 7.1), and 44.8% were boys. In the overall sample, 1.7% of children had functioning difficulties, 28.2% lived in very poor households, 39.4% of mothers had complete middle school, and 50% were between 20–29 years.

**Table 1:** Individual characteristics for the children in the sample.

	Total (n = 2,194)
	% (95% CI)
Functional difficulties	
* No*	98.3 (97.6, 98.8)
* Yes*	1.7 (1.2, 2.4)
Maternal education	
*None*	2.0 (1.3, 3.0)
*Primary*	14.1 (11.4, 17.3)
*Middle school*	39.4 (33.5, 45.7)
*High school*	23.0 (19.4, 27.1)
*University*	21.5 (13.3, 33.0)
Wealth quintiles for urban households	
*Very poor*	28.2 (23.3, 33.8)
*Poor*	21.9 (17.9, 26.6)
*Middle*	18.8 (15.3, 22.9)
*Rich*	21.7 (13.5, 32.9)
*Very rich*	9.4 (6.9, 12.6)
Maternal age	
*15–19 yr*	2.4 (1.0, 5.3)
*20–24 yr*	23.8 (19.8, 28.4)
*25–29 yr*	26.2 (22.1, 30.9)
*30–34 yr*	25.6 (17.2, 36.2)
*35–39 yr*	13.7 (10.6, 17.5)
*40–44 yr*	6.4 (4.8, 8.6)
*45–49 yr*	1.9 (1.1, 3.2)
Sex	
*Boys*	44.8 (38.5, 51.2)
*Girls*	55.2 (48.8, 61.5)
Children age (months)	
*Mean (SD)*	48.8 (7.05)
Maternal age at birth	
*<20 yr*	14.1 (11.2, 17.6)
*20–34 yr*	74.4 (69.7, 78.7)
*35–49 yr*	11.5 (8.9, 14.7)
Indigenous condition	
* No*	78.15 (73.6, 82.1)
* Yes*	21.85 (17.9, 26.4)

Almost 18% of children in urban areas of Mexico had inadequate ECD (Table 2). The largest inadequacies were found in the literacy-numeracy (74.7%) and socio-emotional (20.8%) domains. Children who presented poor development in socio-emotional and literacy-numeracy domains usually had an overall inadequate ECD.

**Table 2:** Overall early childhood development and early childhood development by domain.

Domain	Total (n = 2,194)
	% (95% CI)
Overall early childhood development	
*Inadequate*	17.4 (14.4, 20.9)
*Adequate*	82.6 (79.1, 85.6)
Socio-emotional	
*Inadequate*	20.8 (17.4, 24.8)
*Adequate*	79.2 (75.2, 82.6)
Physical	
*Inadequate*	1.6 (1.1, 2.3)
*Adequate*	98.4 (97.7, 98.9)
Literacy-numeracy knowledge	
*Inadequate*	74.7 (69.9, 78.9)
*Adequate*	25.3 (21.1, 30.1)
Learning	
*Inadequate*	2.2 (1.5, 3.1)
*Adequate*	97.8 (96.9, 98.4)

Table 3 shows municipality characteristics according to children’s early development status (adequate or inadequate ECD). The number of libraries was lower in municipalities where children with inadequate ECD lived, compared to those where children with adequate ECD lived (10.3 vs. 12.2 libraries, respectively). Municipality population density, and density of population under 5 years old, were also lower in areas where children with inadequate ECD lived. Other characteristics of municipalities were different across ECD status, but they were not significant. For example, children with inadequate ECD lived more frequently in smaller municipalities (municipal surface 1,553.5 km^2^ vs. 1,383.2 km^2^ in the group of adequate ECD). They also lived in areas with a higher marginalization index, while the prevalence of crime, insecurity perception, and governance features in municipalities, were quite similar between both groups (Table 3).

**Table 3:** Neighborhood characteristics for the municipalities of children with inadequate or adequate early childhood development in the sample.

Variables	Inadequate ECD	Adequate ECD	P-value	Total
	mean (SD)	mean (SD)		mean (SD)
**Services**				
Libraries	10.3 (11.08)	12.2 (11.14)	0.01	11.9 (11.19)
Pre-schools	227.3 (217.39)	226.3 (193.2)	0.95	226.4 (197.52)
School population under 5 years	38,077 (37,441)	35,047 (32,525)	0.32	35,573 (33,406)
Daycare centers	76.4 (83.11)	68.1 (67.74)	0.32	69.6 (4.40)
Hospitals and clinics	575.9 (683.47)	525.3 (591.94)	0.51	534.1 (608.27)
**Socioeconomic**				
Marginalization index	-1.14 (0.83)	-1.22 (0.71)	0.24	-1.20 (0.73)
Unemployment rate, %	3.5 (2.1)	3.5 (2.0)	0.76	3.5 (2.0)
Population density (inhabitants per km^2^)	2,153 (4,017)	2,897 (4,431)	0.02	2,767 (4,393)
Density of school population	139 (243)	176 (251)	0.04	170 (251)
under 5 years (inhabitants per km^2^)				
**Physical**				
Municipal surface area (km^2^)	1,383.2 (4,090.8)	1,553.5 (4,332.7)	0.43	1,523.9 (4,309.8)
**Social**				
Perception of insecurity	0.52 (0.18)	0.53 (0.17)	0.25	0.53 (0.17)
Prevalence of crime	0.23 (0.13)	0.24 (0.12)	0.25	0.24 (0.12)
**Governance**				
Spent public budget per capita (Mexican peso, $)	3,334.2 (1,362.5)	3,379.05 (1,153.0)	0.69	3,371.6 (1,188.7)
Administrative sanctions	18.83 (32.66)	26.81 (34.32)	0.12	25.65 (34.68)

Table 4 presents the fully adjusted odds of inadequate ECD associated with municipality-level characteristics. A one standard deviation higher availability of libraries was associated with 45% lower odds of inadequate ECD (OR 0.55; 95%CI 0.43, 0.72). Higher availability of libraries was also associated with 34% lower odds of inadequate socio-emotional development (OR 0.66; 95%CI 0.51, 0.85). A one standard deviation higher availability of daycare centers was associated with 44% lower odds of inadequate literacy-numeracy development (OR 0.56; 95%CI 0.32, 0.97), while a one standard deviation higher availability of hospitals and clinics was associated with 87% higher odds of inadequate literacy-numeracy development (OR 1.87; 95%CI 1.29, 2.72). A higher index of marginalization was associated with 80% higher odds of inadequate learning (OR 1.80; 95%CI 1.06, 3.03). Higher municipality population density was associated with higher odds of inadequate ECD in general and in the learning domain. Other municipality characteristics did not show significant associations with overall ECD, or with other domains under study.

**Table 4:** Adjusted associations between overall inadequate early childhood development, domains, and urban characteristics.

Municipal characteristics	Early child development**		Socio-emotional**		Physical**		Literacy-numeracy**		Learning**	
	OR	95%CI	OR	95%CI	OR	95%CI	OR	95%CI	OR	95%CI
Availability of pre-schools***							1,757	0.614–5.030		
Availability of libraries***	0.554*	0.430–0.715	0.655*	0.506–0.849						
Availability of daycare centers***							0.557*	0.320–0.970		
Availability of hospitals and clinics***							1.873*	1.290–2.719		
Marginalization index									1.795*	1.064–3.028
Population density****	1.011*	1.006–1.016	1.009*	1.004–1.015			0,992	0.971–1.013	1.008*	1.002–1.013
Prevalence of crime									2,391	0.149–38.35
MOR										
Null model	2.050	1.626–2.473	2.270	1.827–2.713			2.495	2.020–2.969	1.939	1.158–2.720
Full model	1.896	1.560–2.232	2.112	1.763–2.461			2.585	2.018–3.152	1.001	1.000–1.001

* p-value <0.05.

** All models adjusted by the set of individual variables of each child: Sex, age in months, functional disability, maternal education, wealth quintiles for urban households, indigenous condition, and maternal age.

*** Re-scaled using z-scores, so that 1-unit change represents one standard deviation change in the services of municipalities.

**** Per km^2^.

Table 4 also shows the results of the Median Odds Ratio; from the null model, it was estimated that, if a child moved from a municipality with a low prevalence of inadequate ECD to one with a higher prevalence, the median increase in the odds of inadequate ECD would be two-folded (MOR = 2.05, 95% CI: 1.63–2.47). This suggests a substantial heterogeneity across municipalities. After individual and city-level variables in the analysis were accounted for, the MOR decreased to 1.90 (95% CI: 1.56–2.23).

## Discussion

In this study, we assessed the association between urban environment characteristics and inadequate overall (and by domain) ECD. We found that greater availability of libraries and daycare centers in Mexican municipalities was associated with lower odds of inadequate ECD. These area-level characteristics were also associated with lower odds of inadequate child socio-emotional development and lower odds of inadequate literacy-numeracy development, independently from household, maternal, and individual characteristics. These findings suggest that the urban environment may be important for ECD in Mexico.

We based our analysis on Goldfeld’s model with important limitations due to data availability. Still, out of five domains, we found associations for two: services and socioeconomic environments. The provision of services includes all resources that are made available to children; in our case, we explored libraries, daycare centers, hospitals, and clinics. Our findings showed an association between higher availability of daycare centers and libraries and lower odds of inadequate socio-emotional and cognitive development. These findings are consistent with a recent meta-analysis that showed daycare centers provide quality stimulation and parental support, which in turn has been linked to better language and cognitive development [11]. A greater availability of libraries may also be associated with access to books, a space for caregivers to read to their children, and cultural activities for children and their families. Literacy programs that may be promoted by libraries are effective to promote literacy-numeracy and socioemotional development [36, 37]. A higher number of hospitals and clinics was associated with higher odds of inadequate literacy-numeracy development. Prior studies found that access to health services [38, 39], as well as proximity to services [40, 41] were associated with adequate ECD, which does not concur with our findings. This may be explained by the fact that municipalities with greater availability of clinics and hospitals, could concentrate a higher number of children with special care needs (such as children with disabilities) who benefit from living in areas where they can find complex medical attention and support to their needs.

We evaluated two indicators of socioeconomic conditions in the urban environment, the marginalization index and the unemployment rate. We found that living in a municipality with a higher marginalization index was associated with higher odds of inadequate development in the learning domain; no association was observed with unemployment rate. Some studies suggest that neighborhood poverty is directly related to worse early language and cognitive development [42, 43]. Likewise, a recent study reported that a large percentage of children do not reach adequate development in regions of the world where extreme poverty remains high [44]. Area-level marginalization could affect the stimulation and support of an adequate ECD, due to child malnourishment and lower access to recreational and educational resources and services.

Additionally, our findings showed that living in a municipality with a high population density is associated with higher odds of inadequate ECD in general and for the literacy domain, a variable that we considered part of the physical environment as an indicator of urban size. Higher population density is commonly associated with more traffic, crime, distress, and fewer opportunities to explore the public space [45]. A particular feature of Mexican cities is that higher population density is also associated with greater urban poverty density and people living in overcrowded conditions. Although bigger cities can bring more opportunities for learning and recreation for children, some studies found higher rates of behavioral problems in children in heavily populated areas than in less densely populated neighborhoods [46, 47]. Factors such as lack of safety and neighborhood social disorder could influence the children’s socio-emotional and cognitive development [48–51].

We did not find an association between the public budget spent by the municipalities and ECD. This might be because our governance measure fails to capture whether budget was allocated to developing and improving child resources. With child development being part of the Sustainable Development Goals, many LMIC (including Mexico) have advanced their child protection agenda [52]. In 2014, a new constitutional law was approved to protect the rights of children, and the federal government created the National Integral Protection System of Children and Adolescents (SIPINNA, for its Spanish acronym). Its purpose is to ensure the implementation of the Convention of the Rights of the Child signed and ratified by Mexico [53, 54]. While our results do not show a significant association between governance and ECD, these political advances create momentum for policymakers to promote local changes where our results would be informative for improving children´s lives.

There are some limitations to our study. The characteristics of the urban environment were measured at the municipal level, which was the geographical level that was publicly available for ENIM. Some municipalities are fairly large and heterogeneous and our study precludes from identifying within-municipality characteristics associated with ECD. However, the findings described are still relevant for public policy, since the elected governments of the municipalities are responsible for the basic public services of their population [55]. We measured the availability of services (e.g., area-level number of libraries), a measure commonly used in urban health studies [56], but we were unable to consider the ability of people to reach those services (i.e., accessibility). One dimension of accessibility could be measuring the distance or travel time from children’s households to certain facilities relevant to ECD [57], however, location information could not be obtained from ENIM. Goldfeld’s model describes a number of variables that could influence ECD, and that were not available at the time of the study to be included in our analysis; we used proxies to account for this limitation, yet, future studies should strive to include a wider range of urban context variables to better understand their potential influence over ECD. Finally, although our cross-sectional study design does not allow to establish causal relationships between urban environment and ECD, it is a first approach to explore potential associations between urban environment domains and ECD.

To our knowledge, this is the first study in Mexico examining associations between urban characteristics and ECD. We provided evidence of the potential benefits that the availability of municipal services may bring to ECD. Local governments concerned to improve child development as part of advancing in the Sustainable Development Goals need to consider the impact urban interventions could have on improving ECD in children living in cities. Orienting policies and community actions to improve the urban environment, such as daycare centers and libraries could be an initial approach to integrate upstreaming determinants of ECD.

## Supporting information

S1 Table Exposure variables. (PDF)
