# Supplementary material for: Early childhood development and urban environment in Mexico
Source: PLoS One. 2021 Nov 17;16(11):e0259946. doi: 10.1371/journal.pone.0259946 (PMC8598011; doi:10.1371/journal.pone.0259946)
Supplement: S1 Table — (PDF) [file pone.0259946.s001.pdf]

**Variable**	**Definition**	**Domain**	**Source**	**Year**
Municipal area	Surface area of the municipality in km^2	Physical	National Geostatistical Framework	2018
Prevalence of crime	Proportion of the population who were victims of crime	Social	National Survey of Victimization and Perception of Public Safety (ENVIPE)	2016
Perception of insecurity	Percentage of the population aged 18 and over who feel insecure in the municipality	Social	National Survey of Victimization and Perception of Public Safety (ENVIPE)	2016
Libraries, daycare centers, and hospitals and clinics	Number of libraries daycare centers, and hospitals and clinics.	Services	National Statistical Directory of economic units (DENUE)	2018
Pre-school centers	Number of schools for children under 5 years of age.	Services	Census of Schools, Teachers, and Students of Basic and Special Education (CEMABE)	2013
School population under 5 years	Proportion of the population between 3 and 5 years of age who attended school	Services	Intercensal Survey	2015
Marginalization index	Multidimensional indicator that measures deprivation on four domains education, housing quality, distribution of the population, and income	Socioeconomic	National Population Council (CONAPO)	2010
Unemployment rate	Proportion of unemployed people as a	Socioeconomic	National Occupation and	2017

	percentage of the labor force		Employment Survey	
Population density	Measurement of population per unit area (km^2)	Socioeconomic	National Institute of Statistics and Geography (INEGI)	2015
Density of school population under 5 years	Measurement of population per unit area (km^2)	Socioeconomic	National Institute of Statistics and Geography (INEGI)	2015
Spent public budget per capita	The amount of per capita budget exercised by the Municipal or Delegational Public Administrations during 2016.	Governance	National Census of Transparency, Access to Public Information and Protection of State Personal Data	2016
Administrative sanctions	Total of sanctions applied by type of sanction to the obligated subjects. (administrative negligence, failure to declare patrimonial assets, violation of laws and nepotism).	Governance	National Census of Municipal and Delegation Governments	2017
